# Stakeholders Want a Menu of Choices: Findings from a Consultation Workshop on Improving Access to Secondary Prophylaxis of Rheumatic Fever and Rheumatic Heart Disease

**DOI:** 10.5334/gh.1555

**Published:** 2026-05-18

**Authors:** Thel K. Hla, Stephanie L. Enkel, Joseph Kado, Elizabeth Eadie-Mirams, Rosemary Wyber, Jonathan Carapetis, Laurens Manning

**Affiliations:** 1 Wesfarmers Centre for Vaccines and Infectious Diseases, The Kids Research Institute, Nedlands, WA, Australia; 2 Medical School, University of Western Australia, Crawley, WA, Australia; 3 Department of Infectious Diseases, Fiona Stanley Hospital, WA, Australia; 4 Yardhura Walani, Australian National University, ACT, Australia; 5 Infectious Diseases Department, Perth Children’s Hospital, WA, Australia

**Keywords:** benzathine benzylpenicillin (BPG), penicillin, rheumatic heart disease, rheumatic fever, secondary prophylaxis, consumer perspective, stakeholder perspective

## Abstract

Intramuscular (IM) injection of benzathine benzylpenicillin G (BPG) forms the cornerstone of acute rheumatic fever (ARF) and rheumatic heart disease (RHD) secondary prophylaxis. BPG is available as either a low-cost powdered formulation or a costlier pre-filled suspension. Most of the global RHD burden lies in low- and middle-income countries, which rely on the powdered formulation. Prospects of achieving RHD control targets continue to be hindered by global BPG supply issues. Guided by a preliminary consultation of global stakeholders published in 2016 which outlined the desired characteristics and changes to the existing BPG product, much progress has been made in improving tolerability of BPG for ARF/RHD secondary prophylaxis. A second stakeholder consultation of experts and consumer representatives was convened during the World Congress on Rheumatic Heart Disease in Abu Dhabi, UAE, in November 2023 with the objective of evaluating alternate delivery models and the need for new BPG formulations. Topics explored included current barriers and enablers to local secondary prophylaxis uptake, desired changes to the delivery model and ways to improve access to secondary prophylaxis in resource-limited settings. Our findings highlight inadequacies of the current one-size-fits-all approach and the need for education, access to health systems and personnel as well as flexibility and adaptability to meet individual and community needs for effective programme delivery while new and improved BPG formulations, which will overcome current barriers, are eagerly awaited.

## Introduction

Regular monthly intramuscular (IM) injections of benzathine penicillin G (BPG) remain the cornerstone, and the preferred first-line approach recommended by the World Health Organization (WHO), for secondary prophylaxis for acute rheumatic fever (ARF) and rheumatic heart disease (RHD) (1). Worldwide, BPG is available in two main formulations: a lyophilised powder that requires reconstitution with sterile diluent prior to injection, or a cold-chain dependent viscous suspension in a pre-filled syringe that is administered directly (2). The former costs <US$1 per dose whilst the latter can cost up to US$470 (3) and is only available in high-income settings such as North America, Australia and New Zealand (2). The majority of the 55 million people affected by ARF/RHD globally live in low- and middle-income countries (LMICs) and remain reliant on the powdered formulation of BPG (4,5,6). In 2013, the World Heart Federation (WHF) committed to a 25% reduction in premature deaths from ARF and RHD among individuals aged <25 years by the year 2025, concurrently endorsing an ambitious target to ensure availability of secondary prophylaxis with high-quality BPG for 90% of the patients with RHD in 90% of high-burden countries to achieve this goal (7). Despite inclusion on the WHO’s Essential Medicines List (8) since its inception in 1977, the global production and procurement landscape of existing BPG formulations mean this target will not be met. Global shortages and stockouts have been driven by a number of factors. There are only four manufacturers of the active pharmaceutical ingredient and only one of these has been approved by a stringent regulatory authority (9,10). Due to low profitability, the production of powdered BPG is only triggered by large minimum orders, leaving little financial incentive for other active pharmaceutical ingredient manufacturers to invest in upgrading facilities due to unreliable demand forecasts (2,11,12).

There are a number of issues with existing formulations of BPG which hamper the implementation of WHO recommended pharmacological secondary prophylaxis for ARF, especially in highly endemic settings (13). Injection of both formulations of IM BPG is associated with significant pain and non-allergic, injection-related adverse reactions (14,15). Powdered formulations are difficult to suspend in the diluent which leads to needle blockage, requiring repeated administrations and added discomfort (2,16). There have been rare instances of fatality following injection with powdered formulations, potentially associated with cardiac compromise in the setting of severe valve disease (17,18). These deaths have informed a recent recommendation for oral penicillin in those with pre-existing severe valvular heart disease in some settings, although this has not been met with universal uptake (19,20). In some contexts, the fear of severe injection-related events among healthcare providers has been identified as one of the critical barriers to successful delivery and overall effectiveness of RHD control programmes (21,22).

The preferred product characteristics identified by a group of global RHD and drug development experts for an alternative long-acting penicillin preparation were published in 2016 (23). This document highlighted the desirability of subcutaneous administration, an extended duration of protection (3–6 months) and cold-chain independence, whilst maintaining affordability (23). In the intervening 8 years, significant progress has been made towards achieving these preferred product characteristics. Phase I and phase II studies have demonstrated that a pre-filled formulation of BPG can be safely delivered subcutaneously for RHD secondary prophylaxis, with less associated pain and discomfort and less frequent dosing (24,25). Subcutaneous administration was found to be preferred by consumers as an alternative delivery option suitable for 10-weekly dosing (24,25,26). Demonstration that subcutaneous delivery is safe and has favourable pharmacokinetics also allows consideration of an additional option of self-administered smaller or ‘mini’ doses on a more frequent basis (e.g. weekly) with the ability to customise dosing according to a participant’s body weight (24,27).

In the context of global supply constraints, the cautious reception to the recent recommendation for oral penicillin in patients with severe valvular disease, recent advances made in subcutaneous delivery of BPG with potential for individual dose customisation and weekly self-administration, and with improved formulations of BPG becoming available in the future which could overcome current barriers, a second stakeholder meeting was convened with the intention of consulting a wider cross-section of stakeholders and experts. The purpose was to guide next steps for alternate models of BPG delivery and to re-evaluate the need and product profile of a new BPG formulation.

## Methods

### Study setting and population

The stakeholders were invited from a convenience sample of people living with RHD, consumer representatives, clinicians, researchers and philanthropists who attended a pre-conference half-day workshop (lasting approximately 5 hours) during the World Congress on Rheumatic Heart Disease 2023, hosted by WHF in Abu Dhabi, United Arab Emirates in November 2023.

### Data collection

Upon arrival and prior to the commencement of formal workshop proceedings, attendees were invited to complete a quantitative survey (supplementary materials) containing 15 questions (3 of which were open-ended), administered electronically by scanning a QR code (Google Forms, USA). The questions related to respondents’ involvement in ARF/RHD work or lived experience, their perceived barriers or enablers to successful delivery of secondary prophylaxis in their local setting and a ‘wish list’ for improving currently available delivery methods of secondary prophylaxis. This was followed by focus groups and a panel-led discussion forum led by senior infectious disease physicians (Researchers JC, LM) exploring similar themes, which was audio recorded with the consent of all attendees.

### Data analysis

Survey responses were compiled for quantitative analysis (Excel® for Microsoft 365 MSO, USA). The audio-recorded panel discussion was transcribed verbatim and, together with free-text survey responses, imported into NVivo (Lumivero, USA) for qualitative analysis using deductive methods by two authors (TH; infectious disease physician and researcher, and SLE; public health mixed-methods researcher). An initial coding framework was scaffolded against the survey questions and workshop objectives (barriers/enablers to secondary prophylaxis delivery; preferred delivery models and desired characteristics of BPG formulations), with inductive codes added where new concepts emerged. LM consulted on initial codes, providing clarification as needed. To align with the WHF focus on improving access to secondary prophylaxis in high-burden, resource-limited settings, we developed and prioritised themes that captured variation in stakeholder roles and settings, with emphasis on constraints and implementation considerations most relevant to low-resource contexts. Illustrative quotes were purposively selected to represent a range of roles and geographies, with emphasis on perspectives describing constraints typical of resource-limited settings. Findings from both the surveys and the panel discussions were combined and reported together.

## Results

The main findings from the consultation according to the stated objectives are summarised in Table 1.

**Table 1:** Summary of main findings from the stakeholder consultation with regards to secondary prophylaxis delivery and profile of BPG formulation.


**Secondary prophylaxis**	**Current model:** Pharmacological prophylaxis: Intramuscular injection of BPG every 3–4 weeks Delivery: By a healthcare worker in a healthcare facility By a healthcare worker in schools/homes

	**Alternate/Preferred model as per stakeholders:** Consumer-centred delivery with careful education to support informed decision making and personal autonomy Pharmacological prophylaxis: Choice of— Intramuscular injection of BPG every 3–4 weeks High dose BPG delivered subcutaneously 10-weekly Small dose BPG self-administered subcutaneously weekly Daily oral penicillin (only in highly selected/special populations) Delivery: Choice of— Self-administration at home Home administration by a trained parent or caregiver By a healthcare worker in a healthcare facility By a healthcare worker in schools/homes

**Profile of BPG**	**Preferred product characteristics of new BPG formulation as previously reported** (23) Dose interval longer than 6 weeks, ideally up to 6 months Less painful than existing formulation or ideally painless Suitable for subcutaneous injection Cold chain independent Comparable or lower than the annual cost of existing formulations and delivery

	**Additional preferred product characteristics of the new BPG formulation reported from this consultation** Quality product meeting rigorous standards (GMP certified) Able to be manufactured in adequate quantities to meet global demands from both high- and low-resource settings Accountable and transparent supply chain (prevent stockouts) Cost-effective within the context of broader health systemic costs (direct and indirect costs) beyond price equivalence per dose to current formulation A ‘cost-plus’ tiered pricing model, prioritising consumers in LMICs to ensure equitable access while not disincentivising manufacturers from quality assured production


Abbreviations: BPG—benzathine benzylpenicillin G; GMP—Good Manufacturing Practice; LMICs—Low- and Middle-income Countries.

### Stakeholder background

Forty stakeholders attended the consultation workshop, representing Australia/Timor-Leste (62.5%), Bangladesh (2.5%), Fiji/Pacific Islands (7.5%), India (2.5%), New Zealand (10%), Pakistan (5%), Sudan & Yemen (2.5%), Switzerland (2.5%) and Uganda (2.5%). The roles of stakeholders in relation to ARF/RHD included clinicians (infectious diseases physicians and cardiologists; 47.5%), consumer representatives (including health advocates and those with lived experience of RHD; 5%), researchers (scientists, programme managers, research support workers, health economists; 40%) and representatives of research funders or philanthropy (5%).

Due to technical issues experienced with internet connectivity by many attendees on the day, only 22 responses to the quantitative survey were received in real-time. Retrospective completion of the survey was not offered given that the survey results guided the panel-led discussions. Half of the respondents (11/22, 50%) indicated presence of both a RHD registry and a control programme in their local setting, while 4/22 (18%) only had a control programme, 1/22 (5%) had a registry only and 3/22 (14%) had neither. Primary BPG formulation used in the respective local settings was the pre-filled formulation for 13/22 (59%) and powdered formulation for 9/22 respondents (41%).

### Delivery of secondary prophylaxis

#### Flexibility in the methods of delivery

The majority of respondents (19/22, 86%) agreed with BPG injections being delivered by healthcare workers. Half (11/22, 50%) felt that, if feasible, patients should be able to self-administer injections, while 9 (40%) advocated for parents being able to deliver them for young children at home. Seventeen (77%) indicated a desire for flexibility in the location where BPG could be delivered, with home, school, work or another location of patient’s choice provided as alternatives to traditional clinic settings. While such flexibility may be considered aspirational in most resource-limited settings, it was identified as an important component of successful delivery in settings with resources to enable it.

‘We have approximately 1000 people in a new program where children and teens at school are offered injections usually at school once a month and followed very closely by community nurses and then there are district nurses who deliver injections to adults at community primary care centres and adherence is excellent for the school aged students’

—Clinician working in New Zealand

‘I put self-administration [as number one] […] that would be something that we would love only because we don’t have to come to the hospitals every month. […] that is the option that we as people living with RHD would take because it solves so many issues other than pain, such as forking out money every month for those who live so far away from health centres’

—Consumer representative and community organisation manager, Fiji

#### Ensuring consumer education

Consumer education was perceived as an enabler when done well, and conversely as a barrier when lacking. Forty-seven percent of survey respondents identified a lack of education or awareness as a barrier to successful secondary prophylaxis delivery while 50% identified consumer and community awareness around ARF/RHD as an enabler of success.

‘One of the things we lack in Fiji is awareness and most of our people living with RHD don’t understand what they have. This is one of the biggest issues of default [loss to follow-up] is adherence rates. Part of the program I do is to default chasing—my role there is to educate our people living with RHD the basics of the heart itself what it looks like and what is wrong, for them to understand that and to also understand what the injection is actually doing, which is very important. Once we give them this information, then they start coming to the health centre.’

—Consumer representative and community organisation manager, Fiji

During the meeting, there was no discussion on strategies to enhance awareness or undertake education campaigns, with attendees recognising that this was not only beyond the remit of the discussion but required a targeted and individualised approach tailored to each community and country represented.

#### Managing pain and adverse injection reactions

Pain and discomfort associated with BPG injections was identified as a major barrier in achieving adequate secondary prophylaxis coverage in ARF/RHD by many attendees. For attendees from LMIC settings which primarily use powdered formulations of BPG, additional concerns included poor solubility leading to frequent needle clogging and the need for repeat injections. Practitioners who have witnessed death from cardiovascular compromise following BPG injections in those with severe valvular heart disease described the impact on communities and anxiety among patients, families and healthcare professionals which threaten the viability of RHD control programmes.

‘Witnessing that [BPG] being given is awful, receiving it is dreadful, and I’ve seen children who have had to have four needles placed for the one administration because it is really horrendous. In the context of that, severe pain and severe underlying RHD, I think prioritising a formulation that can provide less pain is important.’

—Clinician working in Timor-Leste

‘We had five patients who died immediately after the injection. […] If a healthcare worker injects a child who dies, then this healthcare worker will have problems in the future to administer the medication.’

—Clinician working in Sudan and Yemen

Conversely, where pain management strategies are employed at time of secondary prophylaxis delivery—such as co-administration of local anaesthetic agent, use of a Buzzy® device (28) (a vibrating case with an optional ice pack which reduces sharp pain when placed proximal to the site of IM injections) and distraction where available (e.g. using games via electronic devices)—this was perceived to have an enabling effect on engagement and coverage by some attendees. However, the availability of these pain management tools remains restricted to those in well-resourced settings.

#### Other barriers and enablers

Other barriers identified by the survey respondents in their local settings included negative health system factors (such as lack of workforce availability and high staff turnover), poor access to healthcare, poor relationship with healthcare workers, systemic racism, lack of patient empowerment and autonomy, and financial and time cost to patients incurred from attending appointments. Similar themes were reflected in the focus group discussions.

In addition to education, other enablers identified included successful relationships with local healthcare workers, trust in the health system, access to healthcare, having familial support, maintenance of patient autonomy, culturally safe delivery of care and modalities that are associated with less pain and financial cost to the patients.

### Characteristics of the ideal secondary prophylaxis

#### Providing choice through a ‘menu of options’

Over two-thirds of survey respondents (15; 68%) felt that ‘a menu of options’ would be required to meet the diverse needs of secondary prophylaxis delivery across different socio-economic and geographical settings, ensuring that the choices are explained to consumers with careful education to support informed decision making and personal autonomy. The majority supported including choices according to consumer preferences, such as small volumes for weekly self-injection suited to home administration (16, 72%) or longer lasting injections of higher volumes into the subcutaneous tissue delivered in healthcare facilities (19, 86%), in addition to the current standard regimen of IM delivery every 3–4 weeks. In group discussions, many attendees also expressed the need for an oral option on the menu for certain populations—such as those who are unable to attend a healthcare facility regularly for geographical or economic reasons who might otherwise become lost to follow-up, or those at risk of cardiovascular compromise due to severe valvular heart disease.

‘I really am liking the menu option […] People need to sit down with the healthcare worker and be told exactly what the risks and benefits are so that they can be involved in proper shared decision making. Menu idea is good […] but you have to have informed decisions.’

—Health advocate working in Australia

Notwithstanding the historical evidence of daily oral prophylaxis being suboptimal compared with monthly injections, many felt it may still have a role in secondary prophylaxis and, therefore, further efforts should be made to update the evidence of its efficacy with modern pharmacokinetic testing and explore dosing schedules that promote adherence (e.g. once daily).

‘Despite the issues with oral adherence, they will get more antibiotics from oral than injection because they simply won’t turn up for an injection.’

—Clinician working in Timor-Leste

‘We’ve had this real barrier in our thinking against oral medication but if that is what is most important for some patients that will change their experience with ARF and RHD then I think that is a big thing for me as a clinician and researcher to think maybe we should be looking more at that?’

—Clinician/researcher working in Australia.

#### Ensuring affordability and equitable access

When asked to decide what a new and improved BPG formulation should cost per dose to reach cost-effectiveness in their region, 9 (43%) respondents were unsure or did not know, while the remaining 12 responses were equally divided in their opinion that it should cost less than US$1, US$1–5, US$10–20 or <US$20, respectively. In focused discussions, some attendees linked frequent global shortages of BPG, unstable supply and variable quality to its low cost (typically less than US$1/dose), making BPG unprofitable to manufacturers and unsustainable for quality assured production.

‘The details need to be discussed in the context of a global problem of shortage […] there is a problem of the drug itself being manufactured and there is no profit out of this. I think the global institutions have to invest in providing the medication at a global level because the companies won’t profit out of this. […] I don’t think the cost is a big issue.’

—Clinician working in Sudan and Yemen

Another emerging theme was for cost analyses to include the broader systemic costs of secondary prophylaxis beyond price equivalence of BPG per dose. There was an overall willingness expressed for higher prices for a better product, even in resource-limited settings. Some felt an improved formulation that costs more but has a longer duration of efficacy would alleviate issues associated with the current powdered BPG formulation and therefore would be cost-effective through a system-wide benefit (i.e. reduced demand on healthcare workers, improved adherence, reduction in complications and reduced travel costs to patients). Parallels were drawn with other ‘expensive’ therapies that are now available in resource-limited settings.

‘Because it is not a daily injection. Someone’s receiving it once a month or every 3 months. We were just looking at survey data of thrombolytic therapy in Sub-Saharan Africa which goes for about 1000 dollars [per dose], and many countries are providing this for free now. So I think there is definitely a case to be put forward for quality penicillin for the prevention of RHD, at a price that is acceptable to most.’

—Clinician working in Uganda

## Discussion

RHD is a globally significant and preventable cause of cardiovascular morbidity and mortality which disproportionately affects socioeconomically disadvantaged people living in LMICs and certain communities in high-income countries (29). Effective global action to improve delivery and access to BPG requires an acknowledgement of the diverse and unique health landscapes faced by those living with RHD and those working to combat RHD in their local settings. A strength of this stakeholder consultation was the diversity of representation across different roles and geographical regions compared to the initial consultation—including people living with RHD, consumer representatives and funders—who ultimately determine the acceptability of any new secondary prophylaxis approaches.

The first key theme from this consultation was the strong support shown for a consumer-centred ‘menu of options’ for secondary prophylaxis delivery. This underscores the need for flexibility to tailor care to consumers according to regional, community and individual needs and for alternatives to the current one-size-fits-all approach. Providing choice allows secondary prophylaxis to be adapted to individual circumstances to optimise adherence, with customisable options in route of administration (IM, subcutaneous or oral), site of delivery (in healthcare facilities, school, self- or carer-delivered at home) and dosing interval (every 2–3 months, monthly, weekly or daily). New opportunities for BPG delivery have not been available since the 1950s when IM injections became standard of care for RHD secondary prophylaxis. Therefore, recent advances made in alternate modes of subcutaneous delivery or self-administration, and the potential for improved reformulations of BPG becoming available in the future which will overcome current barriers, were met with enthusiasm by attendees of this consultation for whom these changes have been long-awaited (24,25,27). Furthermore, stakeholders identified a clear ongoing role for oral penicillin prophylaxis, while highlighting the need for careful patient selection and a better understanding of its pharmacokinetics to optimise dosing in relation to a known pharmacological correlate of protection.

Patient preferences amongst children and young people receiving regular injectable medications for other conditions accord with some of the findings from the stakeholder meeting. A recent systematic review identified impact on daily life, ease of use, tolerability, agency and factors in health sector delivery as key themes driving patient preferences (30). Additional options for ARF/RHD secondary prophylaxis address many of these themes, by providing flexibility, improved tolerability, promoting personal choice in decision making and minimising impact on daily life through self-administration or less frequent attendance at health centres. Further progress has been made in this regard since the consultation took place. A pilot trial is currently being planned for weekly self-administration of BPG in West Australian children and young adults living with ARF/RHD. New candidate formulations for BPG have been developed and evaluated in pre-clinical benchtop experiments and small animal studies.

A second key theme arising from the workshop was the need to strengthen the global supply chain and improve access to high quality BPG preparations in light of frequent stockouts and unreliable global manufacturing. Supply shortages in recent years have been driven by commercial disincentives, as BPG is a low-cost, off-patent medication with limited profitability and a relatively high manufacturing cost. There are few manufacturers of the active pharmaceutical ingredient, meaning that market-driven approaches fail to guarantee reliable supply. Moreover, the product itself is sub-optimal, coming either in an expensive, pre-packaged and cold-chain dependent liquid formulation affordable only to those in high-income countries, or in a powdered and under-priced form that is notoriously difficult to inject (with needle clogging a major issue) that is used in LMICs.

Beyond changing the route of administration to exploit the favourable pharmacokinetics of subcutaneous administration, significant progress has been made towards a reformulation of BPG to match the target product profile set out in the previous stakeholder meeting (23). Although an implant is technically feasible and results in sustained penicillin concentrations, the size required is too large to be a viable solution (31). As evidenced by the stakeholders’ concerns, securing global supply will require a reconfiguration of the international market with investment in both the production of the active pharmaceutical ingredient to Good Manufacturing Practice standards, and the development of a new pre-filled formulation with stringent regulatory authority approvals. These approvals, together with WHO pre-qualification will provide an opportunity for procurement at scale. Interestingly, the stakeholders were uncertain about the optimal price-point but noted that unit pricing for any new formulation would not necessarily need to match the current costs for some powdered formulations (US$ 0.2–0.4).

Since this consultation took place in 2023, the changing geopolitical landscape and shifts in global health funding have further exacerbated threats to the BPG supply chain. In 2025, the United States adopted international reference pricing and Most-Favoured-Nation (MFN) pricing policy, which may incentivise many pharmaceutical companies to withhold supply to smaller markets (such as Australia and New Zealand) or cease supplying LMICs altogether to avoid lowering prices (32,33,34). An alternative to the current profit-driven model is therefore urgently needed. The manufacture of current and future formulations of BPG should instead operate under a ‘cost-plus’ tiered pricing model, which prioritises procurement for people living in LMICs while maintaining incentives for industry innovation.

There were limitations in this consultation process. Stakeholders were sampled by convenience from people already planning to attend the RHD Congress in Abu Dhabi. This meant that the relative proportion of attendees from LMIC settings, and of those living with ARF/RHD was low when compared with clinicians and researchers from high-income settings. Secondly, despite the organisers’ best efforts and due to internet connectivity issues, some attendees were unable to log on and complete the survey resulting in a low response rate. However, all attendees participated in the panel-led discussion which was recorded and transcribed.

## Conclusion

Stakeholders in this study, comprising people living with RHD, consumer representatives, clinicians, researchers and philanthropists, are calling for the development of a consumer-centred ‘menu of options’ as the most promising approach to improving delivery and uptake of BPG as secondary prophylaxis to prevent RHD. In parallel, they highlight the need for an improved formulation with the appropriate stringent regulatory approvals, ideally in a pre-filled syringe which is less painful, administered subcutaneously and priced somewhere between existing powdered and pre-filled preparations.

## Additional File

The additional file for this article can be found as follows:

10.5334/gh.1555.s1 Supplementary Materials. Next generation long-acting penicillins: key features and the global landscape.
