# Supplementary material for: Stakeholders Want a Menu of Choices: Findings from a Consultation Workshop on Improving Access to Secondary Prophylaxis of Rheumatic Fever and Rheumatic Heart Disease
Source: Glob Heart. 2026 May 18;21(1):39. doi: 10.5334/gh.1555 (PMC13196686; doi:10.5334/gh.1555)
Supplement: Supplementary Materials. — Next generation long-acting penicillins: key features and the global landscape. [file gh-21-1-1555-s1.pdf]

Next generation long-acting penicillins: key features and the global landscape

[redacted]

1. In relation to ARF/RHD, which country/region do you work/research?

Your answer

2. Is there a national RHD control program and/or RHD registry in your country or region?

- [ ] Registry
- [ ] Control Program
- [ ] Nil

3. Which of the following apply to you:

- [ ] Clinician
- [ ] Researcher
- [ ] Living with RHD (or family member)
- [ ] Philanthropy
- [ ] Program support
- [ ] Other:

4. Which penicillin preparation is most used in your country/region (in answer 1 above) for RHD secondary prophylaxis?

Choose

5. Who should deliver BPG injections?

- [ ] Health care workers
- [ ] Parent
- [ ] Self/ patient
- [ ] No preference
- [ ] Other:

6. Where should BPG be delivered?

- [ ] Healthcare facility
- [ ] Home
- [ ] School
- [ ] Work
- [ ] No preference
- [ ] Other:

7. What would be the ideal injection choice (s):

- [ ] miniSCIP: Once a week (with smaller volume; suited to self or home administration)
- [ ] very 3-4 weeks (similar volume)
- [ ] SCIP, 4 or 5 times a year (higher volume, clinic delivery)
- [ ] All of the above – a menu of options

8. Do you think that miniSCIP (i.e weekly self-administration of a small volume, less painful injection) would be acceptable in your setting?

Choose

8(a). If you answered no in the above question, then why?

Your answer

9. Do you think that SCIP (i.e a higher volume delivered subcutaneously in a healthcare facility) would be acceptable in your setting?

Choose

9(a). If you answered no in the above question, then why?

Your answer

10. Beta-Pen may cost more than existing products such as powdered BPG. At what cost per dose would Beta-Pen be cost-effective in your region?

Choose

11. In the country/region where you work, who/which would be the regulatory body responsible for approving Beta-Pen?

Your answer

12. Most countries have a cold-chain set-up for vaccine delivery. Should the new product rely on the cold chain?

Choose

13. From your perspective, what do you believe are the greatest barriers to successful treatment delivery?

Your answer

14. For those that successfully engage with treatment, from your perspective, what are the greatest enablers for these patients.

Your answer

15. Do you have any further suggestions regarding how to improve BPG formulations?

Your answer

Submit Clear form

Never submit passwords through Google Forms.

This content is neither created nor endorsed by Google. Report Abuse - Terms of Service - Privacy Policy

Google Forms

Completed COREQ checklist

COREQ (COnsolidated criteria for REporting Qualitative research) Checklist

A checklist of items that should be included in reports of qualitative research. You must report the page number in your manuscript where you consider each of the items listed in this checklist. If you have not included this information, either revise your manuscript accordingly before submitting or note N/A.

Topic	Item No.	Guide Questions/Description	Reported on Page No.
**Domain 1: Research team and reflexivity**			
Personal characteristics			
Interviewer/facilitator	1	Which author/s conducted the interview or focus group?	Pg 6
Credentials	2	What were the researcher's credentials? E.g. PhD, MD	Pg 6
Occupation	3	What was their occupation at the time of the study?	Pg 6
Gender	4	Was the researcher male or female?	NA
Experience and training	5	What experience or training did the researcher have?	Pg 6
Relationship with participants			
Relationship established	6	Was a relationship established prior to study commencement?	Pg 5
Participant knowledge of the interviewer	7	What did the participants know about the researcher? e.g. personal goals, reasons for doing the research	NA
Interviewer characteristics	8	What characteristics were reported about the inter viewer/facilitator? e.g. Bias, assumptions, reasons and interests in the research topic	NA
**Domain 2: Study design**			
Theoretical framework			
Methodological orientation and Theory	9	What methodological orientation was stated to underpin the study? e.g. grounded theory, discourse analysis, ethnography, phenomenology, content analysis	Pg 6
Participant selection			
Sampling	10	How were participants selected? e.g. purposive, convenience, consecutive, snowball	Pg 5
Method of approach	11	How were participants approached? e.g. face-to-face, telephone, mail, email	Pg 5
Sample size	12	How many participants were in the study?	Pg 7
Non-participation	13	How many people refused to participate or dropped out? Reasons?	Pg 7
Setting			
Setting of data collection	14	Where was the data collected? e.g. home, clinic, workplace	Pg 6
Presence of non-participants	15	Was anyone else present besides the participants and researchers?	NA
Description of sample	16	What are the important characteristics of the sample? e.g. demographic data, date	Pg 6
Data collection			
Interview guide	17	Were questions, prompts, guides provided by the authors? Was it pilot tested?	Table 1
Repeat interviews	18	Were repeat inter views carried out? If yes, how many?	NA
Audio/visual recording	19	Did the research use audio or visual recording to collect the data?	Pg 7
Field notes	20	Were field notes made during and/or after the inter view or focus group?	Pg 6 - transcrib
Duration	21	What was the duration of the inter views or focus group?	Pg 6
Data saturation	22	Was data saturation discussed?	NA
Transcripts returned	23	Were transcripts returned to participants for comment and/or	NA

Topic	Item No.	Guide Questions/Description	Reported on Page No.
		correction?	
**Domain 3: analysis and findings**			
Data analysis			
Number of data coders	24	How many data coders coded the data?	Pg 7
Description of the coding tree	25	Did authors provide a description of the coding tree?	NA
Derivation of themes	26	Were themes identified in advance or derived from the data?	Pg 7
Software	27	What software, if applicable, was used to manage the data?	Pg 7
Participant checking	28	Did participants provide feedback on the findings?	NA
Reporting			
Quotations presented	29	Were participant quotations presented to illustrate the themes/findings? Was each quotation identified? e.g. participant number	8, 9, 12, 13, 14
Data and findings consistent	30	Was there consistency between the data presented and the findings?	8, 9, 12, 13, 14
Clarity of major themes	31	Were major themes clearly presented in the findings?	NA
Clarity of minor themes	32	Is there a description of diverse cases or discussion of minor themes?	NA

Developed from: Tong A, Sainsbury P, Craig J. Consolidated criteria for reporting qualitative research (COREQ): a 32-item checklist for interviews and focus groups. *International Journal for Quality in Health Care*. 2007. Volume 19, Number 6: pp. 349 – 357

Once you have completed this checklist, please save a copy and upload it as part of your submission. DO NOT include this checklist as part of the main manuscript document. It must be uploaded as a separate file.
